# Nanoparticle-based delivery enhances anti-inflammatory effect of low molecular weight heparin in experimental ulcerative colitis

**DOI:** 10.1080/10717544.2017.1324530

**Published:** 2017-05-16

**Authors:** Tawfek Yazeji, Brice Moulari, Arnaud Beduneau, Valentin Stein, Dirk Dietrich, Yann Pellequer, Alf Lamprecht

**Affiliations:** 1 Department of Pharmaceutics, University of Bonn, Bonn, Germany,; 2 FDE EA4267University of Burgundy, Besançon, France,; 3 Institute of Physiology II, Medical Faculty, University of Bonn, Bonn, Germany, and; 4 Department of Neurosurgery, University of Bonn, Bonn, Germany

**Keywords:** Ulcerative colitis, LMWH, nanoparticles, polymethacrylate, macrophages

## Abstract

Epithelial administration of low molecular weight heparin (LMWH) has proven its therapeutic efficiency in ulcerative colitis (UC) but still lacks of a sufficiently selective drug delivery system. Polymeric nanoparticles were used here not only to protect LMWH from intestinal degradation but also to provide targeted delivery to inflamed tissue in experimental colitis mice. LMWH was associated with polymethacrylate nanoparticles (NP) type A (PEMT-A) or type B (PEMT-B) of a size: 150 nm resulting in a maximum drug loading: 0.1 mg/mg. In a lipopolysaccharide-stimulated macrophages both, free LMWH and LMWH-NP have significantly reduced the cytokines secretion independently from cellular uptake. The *in-vivo* therapeutic efficiency was dose dependent as at low doses (100 IU/kg) only minor differences between free LMWH and LMWH-NP were found and the superiority of LMWH-NP became prominent with dose increase (500 IU/kg). Administration of LMWH-NP at 500 IU/kg has markedly improved the clinical activity as compared to LMWH while similarly pathophysiological indicators revealed increased therapeutic outcome in presence of NP compared to LMWH alone: Myeloperoxidase (Colitis control: 10 480 ± 5335, LMWH-PEMT-A NP: 1507 ± 2165, LMWH-PEMT-B NP: 382 ± 143, LMWH: 8549 ± 5021 units/g) and tumor necrosis factor: (Colitis control: 1636 ± 544, LMWH-PEMT-A NP: 511 ± 506, LMWH-PEMT-B NP: 435 ± 473, LMWH: 1110 ± 309 pg/g). Associating LMWH with NP is improving the anti-inflammatory efficiency of LMWH *in-vivo* by its protection against degradation in luminal environment and selective drug delivery. Such a combination holds promise for a highly specific therapy by its double selectivity towards the inflamed intestinal tissue. LMWH-PEMT NP have significantly improved the clinical activity *in-vivo* in comparison to free LMWH.

## Introduction

Ulcerative colitis (UC) and Crohn’s disease are the predominant forms of inflammatory bowel diseases (IBD) defined as a progressive inflammation in the intestinal mucosa with functional irregularities in the epithelium and mucosal immune system (Podolsky, 2002; Kaser et al., 2010). The mainstay of UC therapy is based on oral or rectal administration of steroid or non-steroids anti-inflammatory drugs. However, a specific delivery and deposition of these drugs at the site of inflammation is still challenging (Lamprecht, 2015).

Recent advances in drug delivery to the inflamed colon tissue in UC have highlighted the potential role of nanoparticles (NP) as drug carriers for passive targeting of the inflamed tissues in the colon. Selective bioadhesion of NP to the inflamed mucosa permits this high selectivity in drug deposition (Lamprecht et al., 2001; Nakase et al., 2001) and additionally NP can enhance intra-tissue penetration of the associated drug cargo (Lai et al., 2009; Lamprecht et al., 2005).

Among a wide arsenal of drugs currently studied for their efficiency in UC, low molecular weight heparin (LMWH) has demonstrated a strong therapeutic potential due to its anti-inflammatory characteristics (Törkvist et al., 1999; Dotan et al., 2001; Vrij et al., 2001; Yang et al., 2001; Chande et al., 2010). However, due to its hemorrhagic adverse effects after systemic availability, a broad clinical use in IBD therapy was impossible. First attempts by epithelial delivery of LMWH elucidated the potential of this therapeutic approach benefitting of low systemic bioavailability and subsequent minimal adverse effect levels (Pellequer et al., 2007; Celasco et al., 2008; Luo et al., 2010).

Although these epithelial LMWH delivery strategies are very interesting owing to their potential in suppressing systemic adverse effects, the drug availability is rather nonspecific due to the classical formulation approaches proposed, e.g. matrix tablets, suppositories or coated capsules (Celasco et al., 2008,2010; Luo et al., 2010,2011). Subsequently, a further improvement of the therapeutic efficiency was therefore postulated by associating LMWH to a nanoscale drug carrier allowing for a more selective targeting and delivery to the inflamed tissue.

Nanoparticles have been investigated here as potential carriers of LMWH to provide a higher specificity and local drug deposition towards inflamed intestinal tissues knowing that they may contribute strongly to LMWH stability in the harsh intestinal environment. However, NP have been observed to preferentially accumulate in immune related cells in the inflamed colonic tissue (Niebel et al., 2012), one major aspect to take into consideration was a potential intracellular uptake of LMWH into macrophages and the potential alteration in drug efficiency.

Accordingly, LMWH was associated with cationic NP prepared from polyethyl acrylate-co-methyl methacrylate-co-trimethylammonioethyl methacrylate chloride (PEMT-NP; Eudragit® RS and RL) to obtain LMWH-loaded NP (LMWH-PEMT-NP). NP formulations were analyzed in terms of their physiochemical characteristics and studied *in vitro* for their cell binding and uptake behavior on macrophages and related consequences for their therapeutic efficiency. Finally, *in vivo* studies were performed on a murine experimental colitis in order to investigate the therapeutic potential of this new delivery system compared to LMWH alone.

## Experimental

### Materials

Polymers used in this study, poly(ethyl acrylate-co-methyl methacrylate-co-trimethylammonioethyl methacrylate chloride) type A (PEMT-A, Eudragit® RL), and type B (PEMT-B, Eudragit® RS), were kindly supplied from Evonik Röhm GmbH (Pharma Polymer, Germany). LMWH, enoxaparin sodium (Clexane® 10 000 IU anti-Xa/ml) was purchased from Sanofi-Aventis (Paris, France). RAW 264.7 cells were obtained from ATCC (Manassas, VA). Lipopolysaccharide (*Escherichia coli* sterotype 0127: B8) was purchased from Sigma Aldrich Chemie GmbH (Steinheim, Germany). Mouse TNF-α, IL1ß, IL6 ELISA kits were purchased from eBioscience® (Sar Diego, CA). All other chemicals and organic solvents were of analytical grade.

### Methods

#### Nanoparticles preparation, characterization, drug association and release

PEMT blank NP were produced according to a modified spontaneous emulsification solvent diffusion method, but using a mixture of acetone/ethyl acetate instead of using acetone/ethanol or acetone/methanol (Murakami et al., 1999). Briefly, 50 mg polymer was dissolved in a mixture of acetone/ethyl acetate (3:4 for PEMT-A, 1:3 for PEMT-B). A fluorescent lipophilic indocarbocyanine dye (DiI) solution in ethyl acetate was added in this step to the organic phase (1 μg DiI/mg polymer) to obtain DiI labeled NP. The polymer solution was then added to 10 ml water without using surfactants. The resulted emulsion was homogenized for one minute using an ultra-sound at 40 W (Sonopuls, Bandelin electronic, Berlin, Germany). Thereafter, the organic solvents were evaporated under low pressure using Büch Rotavapor RE120 (Büchi, Flawil, Switzerland). LMWH was then associated with 50 mg of PEMT blank NP by stirring at 300 rpm/min for 90 min. Finally, LMWH-NP were centrifuged for 30 min at 12 000 rpm using (Hermle Z 233 M-2, Wehingen, Germany) to omit non-associated LMWH. The drug association efficiency was evaluated indirectly using turbidymetric assay as described previously (Demoré et al., 1998). NP were utilized directly in further investigations or lyophilized for drug release studies using a freeze dryer (STERIS Lyovac GT2, Hürth, Germany) as described earlier (Ali & Lamprecht, 2014).

NP formulations were characterized in view of their size, polydispersity index (PDI), shape and surface charge potential using Zetasizer (Brookhaven Instruments, Holtsville, NY). Morphological characteristics of NP formulations were analyzed using a Scanning Electron Microscope SEM (SU3500 Hitachi, Tokyo, Japan).

For *in vitro* drug release tests freeze dried LMWH-NP were redispersed in phosphate buffer at pH 7.4 in presence or absence of mucin (2.5% w/w). Samples were withdrawn at predetermined time intervals, and the drug was determined after filtration using turbidymetric assay. All experiments were run in triplicates, data are presented as means with respective standard deviations.

Unless mentioned otherwise, LMWH-NP were used at the maximum drug/NP association capacity (0.1 mg/mg).

#### Cell culture studies: cell viability, NP localization, inflammatory cytokine secretion, and confocal imaging

RAW 264.7 cells were cultured in Dulbecco’s Modified Eagle’s Medium (DMEM) with fetal calf serum, streptomycin and penicillin. Cells were incubated overnight for cell adherence and split twice a week. All cells were used between passage 8 and 25.

For cytotoxicity tests, cells were incubated for 8 h with the various formulations at different concentrations, then cell viability was determined based on mitochondrial activity (Carmichael et al., 1987) and lethal dose values (LD_50_) were calculated accordingly.

LMWH-NP impact on cytokine release was studied as follows: Macrophages were stimulated with lipopolysaccharide (LPS) 10 μg/ml for 2 h, then LPS-stimulated cells were incubated with the NP for 8 h as described earlier (Niebel et al., 2012). Cytokine levels were determined in cell medium using Mouse ELISA Ready-SET-Go® kits for TNF-α, IL-6 or IL-1β from (eBioscience), according to the manufacturer’s protocols and normalized to protein content in all analyses.

NP were visualized in fixed cells as previously described (Wachsmann et al., 2013) using Confocal Laser Scanning Microscopy (Nikon A1, Nikon, Tokyo, Japan). After incubation with DiI-labeled NP and counterstaining cell membranes with wheat germ agglutinin-FITC (555 nM) at 4 °C, cells fixed and incubated with 4,6-Diamidino-2-phenylindole dihydrochloride (DAPI, 300 nM) for staining of cell nuclei. Besides, in order to trace the drug distribution in the cells, LMWH was labeled with fluoresceinamine according to a previously described procedure (Lamprecht et al., 2006).

#### Animal studies

All animal experiments were performed in accordance with the recommendations in the Guide for the Care and Use of Laboratory Animals (National Research Council, and National Academy of Sciences). Four-week old Swiss albino male mice (average weight: 30 g) were divided into 10 groups, one healthy group and nine colitis groups (*n* = 5).

The colitis groups were divided into groups receiving 100 IU/kg or 500 IU/kg alone or associated with each NP type. One colitis control group was without treatment.

Experimental colitis was induced in mice as previously described (Pertuit et al., 2007). Briefly, 100 μl of Trinitrobenzenesulfonic acid (TNBS) in 50% ethanol was injected intra-rectally (3 cm from the anus, 90 mg/kg body weight). The healthy group received isotonic saline solution instead. 24 hours after colitis induction, mice were treated with LMWH or the respective NP formulations at the same LMWH dose for three days. The colitis control group received isotonic saline solution instead.

A clinical activity score involving animals’ body weight loss, stool consistency and rectal bleeding was recorded daily during the experiment as described previously (Hartmann et al., 2000).

Twenty-four hours after the last administration, all animals were sacrificed and the colon tissues were resected, imaged, minced and underwent biochemical analyses: myeloperoxidase (MPO) activity was evaluated in the tissue samples according to a standard method described previously (Krawisz et al., 1984), giving MPO activity (unit) per wet tissue weight (g), where one unit of MPO activity is referred to as the amount of enzyme that degrades 1 μmol of peroxide in one minute at 25 °C. Cytokine levels for IL-1β, IL-6 and TNF-α were determined in the tissue using ELISA Ready-SET-Go® kits from (eBioscience) according to the manufacturer’s protocols.

#### Statistical analysis

Data are expressed as means ± S.D. Analysis of statistical significance was performed according to Kruskal–Wallis (ANOVA) and followed by Tukey’s test in all pairwise comparisons. Differences were considered significant when *p* ≤ 0.05.

## Results

**Figure 1.**
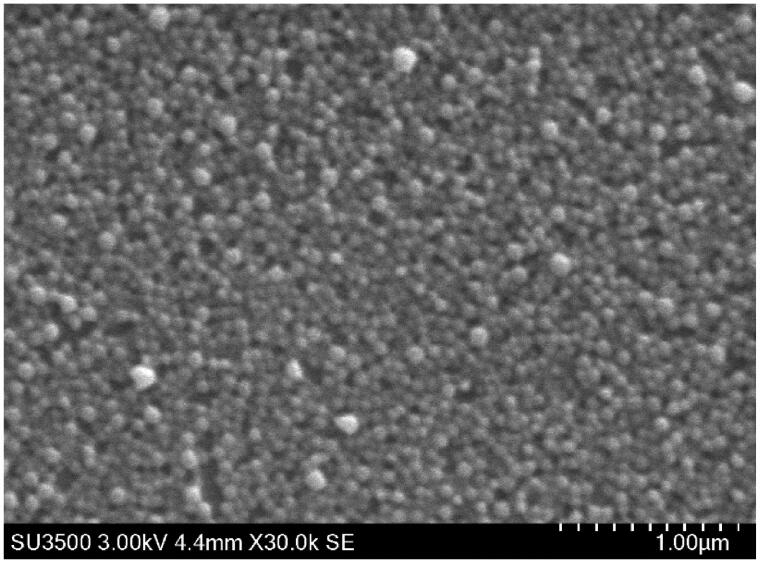
Scanning electron microscope images **(SEM)** of the blank PEMT NP.

PEMT blank NP were spherical (Figure 1) and had a size of around 150 nm with mono-modal size distribution (polydispersity index: 0.14–0.22). The positive surface charge of the blank particles with a zeta potential of about +40 mV remained mainly unchanged at low LMWH loading rates but reversed nearly instantaneously at a theoretical LMWH/NP ratio of around 0.1 mg/mg (10 IU/mg) where the maximum drug association was reached (Supplementary material, figure 1). Both PEMT-A and PEMT-B NP have shown almost identical LMWH association capacities, PEMT-A NP: 0.125 ± 0.024 mg/mg (10.25 ± 2.4 IU/mg), and PEMT-B NP: 0.108 ± 0.008 mg/mg (10.8 ± 0.8 IU/mg).

Slight particle size increase was observed by loading the drug on the blank NP (Supplementary material, figure 2).

**Figure 2.**
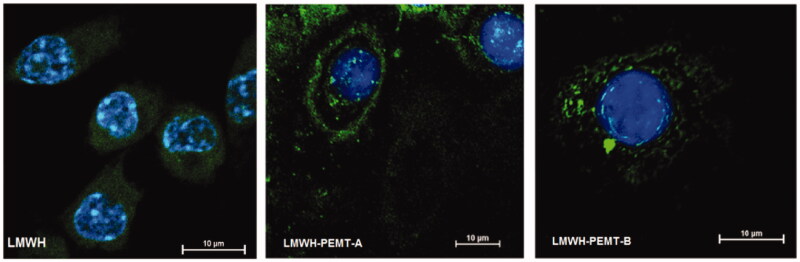
CLSM images showing macrophages with stained nuclei after 2 h incubation with fluorescein labeled LMWH or LMWH-NP.

*In-vitro* drug release studies in phosphate buffer at pH 7.4 revealed a limited drug release from LMWH- PEMT-A NP and - PEMT-B NP with a plateau at around 10 to 20% of the total LMWH load, however, the presence of mucin has triggered the drug release which was nearly completed after 20 h (Supplementary material, figure 3).

**Figure 3.**
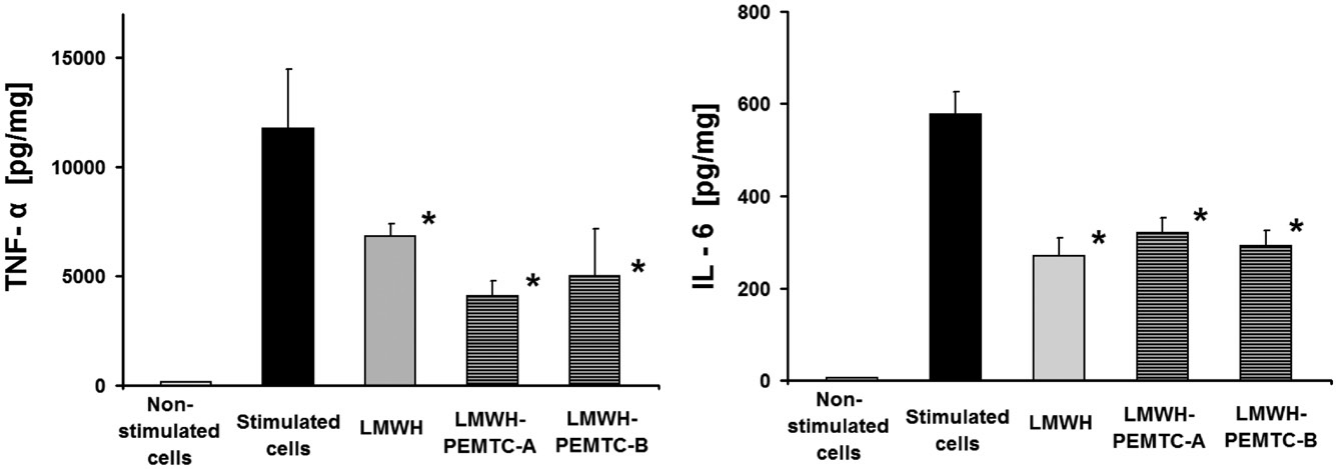
Release of TNF-α and IL-6 from LPS-stimulated macrophages after 8 h of incubation with LMWH (1 IU/ml) and LMWH-NP (0.1 mg/ml). *n* = 3, data represent mean SD. * = *p* < 0.05 compared to stimulated cells.

LMWH and the NP formulations were utilized in cell culture at concentrations that did not influence the cell viability (Supplementary material, figure 4).

**Figure 4.**
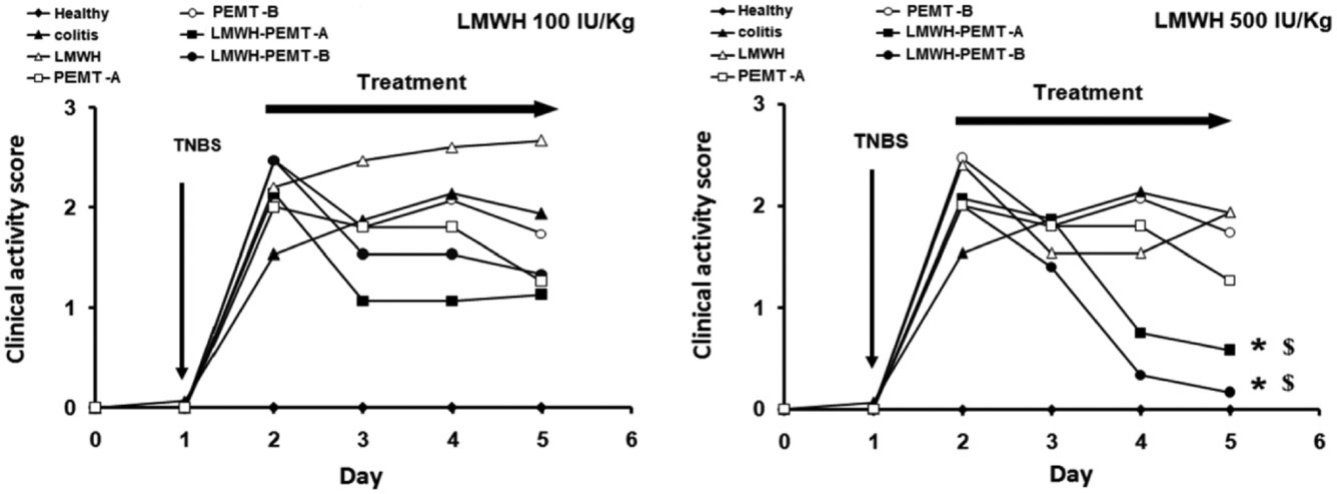
Clinical activity score (CAS) of mice. After colitis induction, the animals were treated for 3 days with rectal injection of blank and LMWH-loaded NP. The drug and LMWH-NP dose were equal to 100 IU/kg (left), or 500 IU/kg (Right). The free drug showed no significant improvement while treatment with LMWH-NP (500 IU/kg) has significantly improved the CAS of colitis mice. *n* = 5, * = *p* < 0.05 compared with colitis control group given saline. $=*p* < 0.05 compared with colitis groups given LMWH (500 IU/kg).

CLSM images have underlined significant uptake and the subsequent NP distribution inside the cytosol. However, NP did not lead to a major change in the intracellular uptake of LMWH (Figure 2). Similarly, no qualitative difference could be observed regarding the distribution of the blank and LMWH-NP (Supplementary material, figure 5).

**Figure 5.**
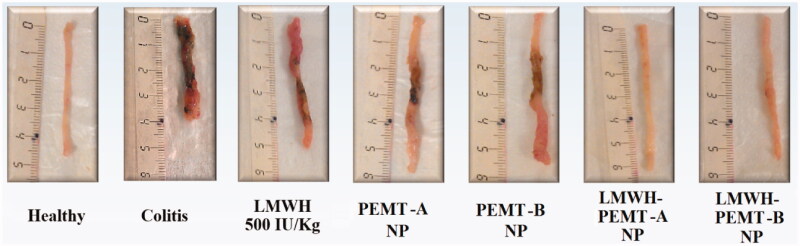
Colon tissues of mice treated with LMWH and LMWH-NP formulations. The dose of both the drug and drug-loaded NP was equal to 500 IU/kg. Neither clear necrosis in the crypt nor an obvious damage was detected in the colonic mucosa of LMWH-NP groups, while clear necrosis was observed in the disrupted colon architecture in the other groups.

LMWH-loaded NP reduced TNF-α and IL-6 release from LPS-stimulated macrophages after 8 h of incubation (Figure 3). Apparently, increasing the drug loading ratio of LMWH/NP did not play a vital role in cytokine release, at least within the tested concentrations of NP (Supplementary material, figure 6).

**Figure 6.**
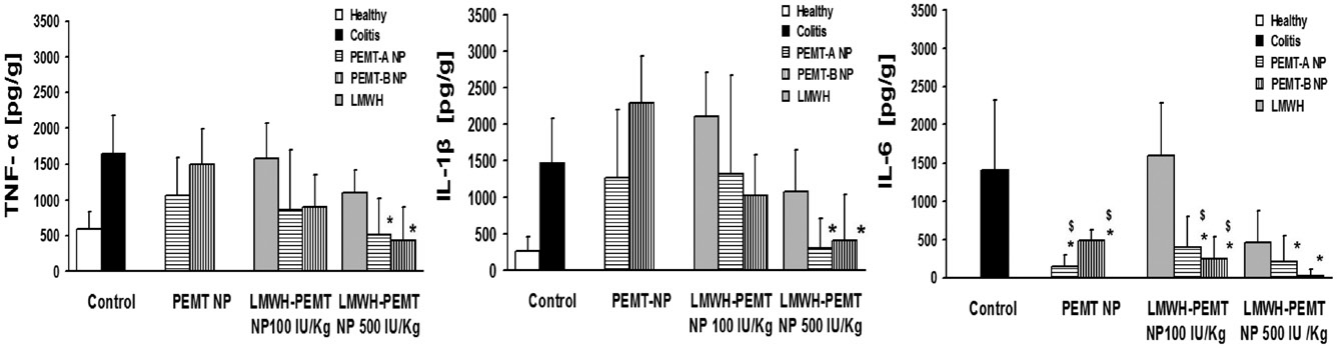
Levels of the inflammatory cytokines in the colon tissue of colitis mice after treatment with LMWH, blank NP and LMWH-NP. Data are shown as mean ± S.D (*n* = 5). *, $= *p* < 0.05 compared to each of the colitis control and LMWH groups respectively. Only LMWH-NP (500 IU/kg) were able to achieve an efficient and significant reduction of TNF-α IL-1β in comparison with colitis control, while LMWH (100 IU/kg) showed no significant efficiency. Both the blank and drug-loaded NP have significantly reduced the levels of IL-6.

*In-vivo*, clinical activity scores did not show any significant benefit of treatments with LMWH formulations at 100 IU/kg. However, a distinct clinical improvement of the inflammation was observed in the LMWH-NP groups at the higher dose of 500 IU/kg, while all other scores at that dose were not significantly different from untreated control (Figure 4). Accordingly, colon tissue samples exhibited significant levels of necrosis and disruption of the intestinal structure throughout (Supplementary material, figure 7). Exceptions were the groups treated with LMWH-NP at 500 IU/kg where tissues had normal appearance and only minor changes in the tissue’s histological manifestation (Figure 5).

MPO levels were not significantly reduced by LMWH or LMWH-NP at 100 IU/kg, but at 500 IU/kg only the two LMWH-NP groups led to a significant efficiency while free LMWH at high dose did not (Supplementary material, figure 8).

Cytokine levels tested in the inflamed tissues showed similar trends. While TNF-α and IL-1β concentrations remained unchanged at high levels at 100 IU/kg, it was repeatedly found that at 500 IU/kg free LMWH failed to show a significant reduction and only LMWH associated to NP led to significant effect (Figure 6). Strongest effects of the therapeutic approach were observed in IL-6 levels, where also LMWH-PEMT NP groups at 100 IU/kg resulted in a significant cytokine reduction and at 500 IU/kg for PEMT-B NP nearly levels of a full remission were observed.

## Discussion

Heparins have been used for anti-inflammatory therapy in IBD with variable success (Michell et al., 2001; Lean, et al., 2015a). A major advantage of oral or rectal administration of heparins is the subsequent local effect combined with minimal systemic availability essentially providing a therapeutic approach without adverse effects (Pellequer et al., 2007). However, all precedent studies were based on systems with limited specificity in delivery (Pellequer et al., 2007; Celasco et al., 2008; Lean, et al., 2015b). NP in the size range of about 100 nm have proven a high potential of adherence to inflamed colon tissues (Lamprecht et al., 2001); therefore, such NP are most suitable in combination with LMWH to achieve a maximum benefit for UC therapy.

The choice of nanoscale delivery systems for local IBD therapy for LMWH was based on the successful association of LMWH onto cationic NP as described earlier (Jiao et al., 2002). A rectal administration was most suitable here in order to evaluate the therapeutic potential of this LMWH-NP concept since based on the experimental design it can be ensured that the entire dose is made available to the site of action. Particles were designed with a mean diameter of around 150 nm being in the proximity of the optimum that has been found earlier for colitis targeting (Lamprecht et al., 2001). The use of surfactants was avoided during particle design in order to allow a maximum LMWH loading capacity which is associated to the particle surface by electrostatic interactions.

The limited drug release in phosphate buffer underlined the advantage of drug**-**loaded NP formulations over the drug alone providing a protective effect to LMWH before reaching the site of action. On the other hand, it became clearly visible that mucus contact of the NP will rapidly lead to a dissociation of LMWH from NP’s surface. This effect was similarly observed in the electrostatically associated anionic molecule (clodronate), where seemingly the polysaccharides and glycoproteins of the mucin molecules dislocated the drug from the cationic surface of NP (Niebel et al., 2012). It is noteworthy that this phenomenon did not reverse the therapeutic effect of the *nano* formulations in neither of the two studies. However, LMWH-PEMT-NP turned out not to be suitable for oral administration without a secondary protective vehicle such as a hydrogel or a capsule.

Since NP tend to accumulate in macrophages, known to be one of the principal mediators of the inflammatory reaction, a suitable strategy seemed to increase the LMWH concentration close to these cells. *In-vitro* cell culture data, however, revealed that although an increased cellular LMWH uptake occurs by the presence of NP, no significant change in inflammation levels is observed in cell culture comparing free LMWH with the NP**-**associated LMWH. Whether this can be directly translated into conclusions on an ineffective increase of intracellular LMWH concentration needs to be seen critically, co-localization of NP and LMWH in CLSM indicated that, in contrast to the release experiments with mucin, no major dissociation of LMWH took place during cell binding and uptake.

The low clinical activity score achieved by treating the mice with LMWH-PEMT-NP, has signified the potential efficiency of these particles in comparison to the blank NP and LMWH alone.

In line with these clinical indicators, the levels of MPO activity which reflect the amount of infiltrated immune cells in the inflamed tissue, have been significantly reduced only at the higher drug dose of LMWH-PEMT-NP. The therapeutic efficiency was further confirmed by the significant reduction of relevant pro-inflammatory cytokines, where again LMWH loading of NP at a high dose led to a significant reduction of the pro-inflammatory cytokines.

Slight discrepancies with another recent study (Pellequer et al., 2007) where no significant changes in TNF-α levels were observed *in vivo* indicate a potentially additional interaction with the inflammatory cascade, which however remains unknown until now.

While in an earlier study the intracellular delivery of clodronate by NP was essential for obtaining a therapeutic effect (Niebel et al., 2012), the interpretation of the *in-vivo* results is more complex in this study.

In view of macrophages being key mediators found in the colon of IBD patients, several studies revealed that M1 versus M2 macrophage subsets play an import role in the course of the disease where M1 macrophages had pro-inflammatory effects in the model colitis, while M2 macrophages were protective (Weisser et al., 2011; Zhu et al., 2014). Oral enoxaparin treatment in experimental colitis reduced numbers of M1 macrophages and elevated numbers of M2 macrophages assumingly by reducing levels of Granulocyte-macrophage colony-stimulating factor (GM-CSF), which contributes in the differentiation of monocytes into pro-inflammatory macrophages during intestinal inflammation (Lean, et al., 2015b).

Since macrophages are a major source of diverse pro-inflammatory cytokines, this change in M1/M2 ratio explains the reduction of multiple other cytokines such as IL-1α, IL-6, IL-10, TNF-α that originated from inflamed colon tissues. Whether the increase in anti-inflammatory efficiency of LMWH-NP over free LMWH was attributed to increased intracellular availability of LMWH *in-vivo* still needs to be analyzed. However, this interpretation would also be in line with the observed reduction of IL-1β levels with NP associated LMWH, as the cytokine is expressed by macrophages in the inflamed colon (McAlindon et al., 1998).

Another explanation could also be a simple dissociation of drug from the NP surface upon arrival at the inflamed area and a subsequent additive anti-inflammatory effect of the two components, NP on the one side, LMWH on the other. This would fit with the assumption that PEMT-A and PEMT-B NP could lead to a “mucin-wrapping” a drug penetration mechanism that has been proposed recently (Wang et al., 2011; Viehof & Lamprecht, 2013), allowing an enhanced penetration of LMWH into the inflamed tissue area. Also, the protection of LMWH against early degradation in the intestinal milieu may play a role, although the impact is probably minor due to the rectal administration directly at the site of inflammation. If enzymatic degradation was an issue in this limited tissue area, earlier studies (Lean et al., 2015b) would not have found an effect after total unprotected transit throughout intestinal tract at all.

The reason as to why the blank NP have also shown an anti-inflammatory property is not clear. This behavior could be related to the impact of the cationic NP on the macrophage’s immune function. For example, it was assumed that positively charged groups in cationic polymers might interact with the free anionic carboxyl groups on the lectin-like domains in TNF-α molecules (Hazemi et al., 2010).

Finally, the therapeutic efficiency of LMWH-NP was clearly drug**–**dose dependent, since that increasing the treatment dosage from 100 to 500 IU/kg has significantly improved the therapeutic efficiency. This should be seen in the context of other studies where oral LMWH were efficient at much higher doses when administered orally at 2000 IU/kg **(**Lean et al., 2015b**),** which also explains the discrepancy to the rather inefficient free LMWH group in this study. In turn, this indicates that NP formulations enable to a significant dose decrease, maintaining the therapeutic effect.

## Conclusion

The anti-inflammatory efficiency of LMWH can be strongly intensified by associating the drug with cationic PEMT NP. Facilitating the entrance of LMWH-NP into macrophages did not seem to lead to a remarkable improvement of LMWH anti-inflammatory efficiency in cell culture however, *in-vivo* NP reinforced the efficiency of LMWH. The current formulation provides an efficient therapeutic approach, which offer a dose reduction compared to earlier enteral LMWH therapies. The exact understanding of the macrophage-NP-LMWH interplay may open additional therapeutic perspectives based on this initial study.

## Supplementary Material

IDRD_Alf_et_al_Supplemental_Content.docx

## Notes

**Supplementary material available online**
