# Supplementary material for: Nanoparticle-based delivery enhances anti-inflammatory effect of low molecular weight heparin in experimental ulcerative colitis
Source: Drug Deliv. 2017 May 16;24(1):811–7. doi: 10.1080/10717544.2017.1324530 (PMC8240985; doi:10.1080/10717544.2017.1324530)
Supplement: IDRD_Alf_et_al_Supplemental_Content.docx [file IDRD_A_1324530_SM5332.docx]

**Supporting information**


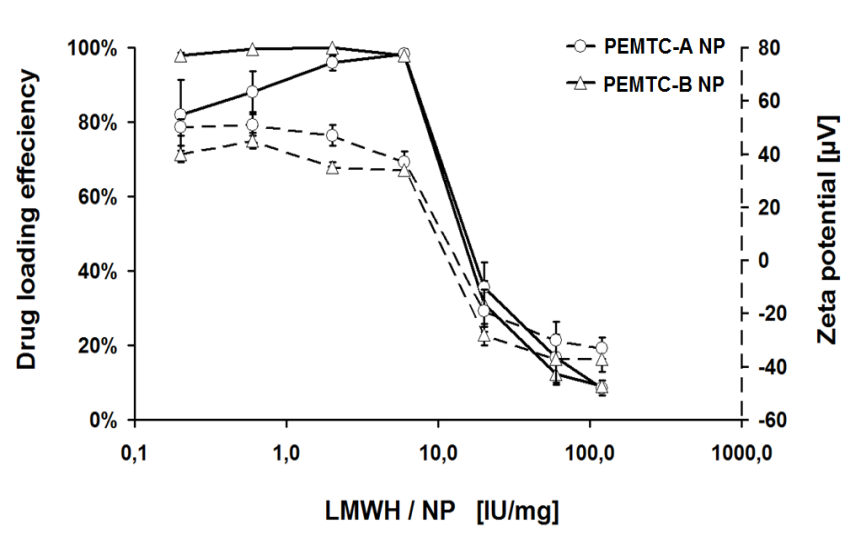


**Figure 1**: Changes in LMWH association efficiency (straight line), and changes in NP’s zeta potential (dotted line) as a function of increasing the drug concentration against fixed particles mass [IU/mg]. n= 3, data represent mean ± SD


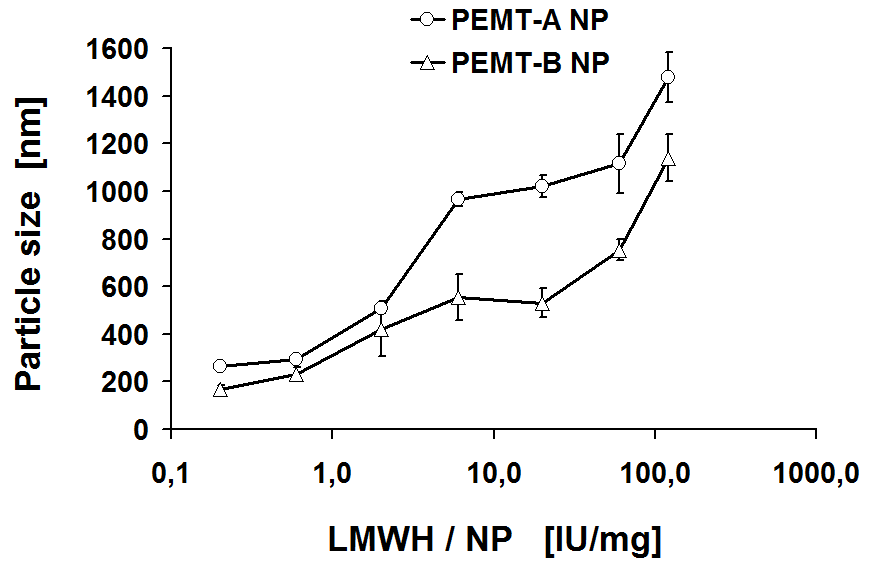


**Figure 2:** The influence of the drug loading on NP’s size. Increased amounts of drug were associated with the blank NP. n= 3, data represent mean SD

**
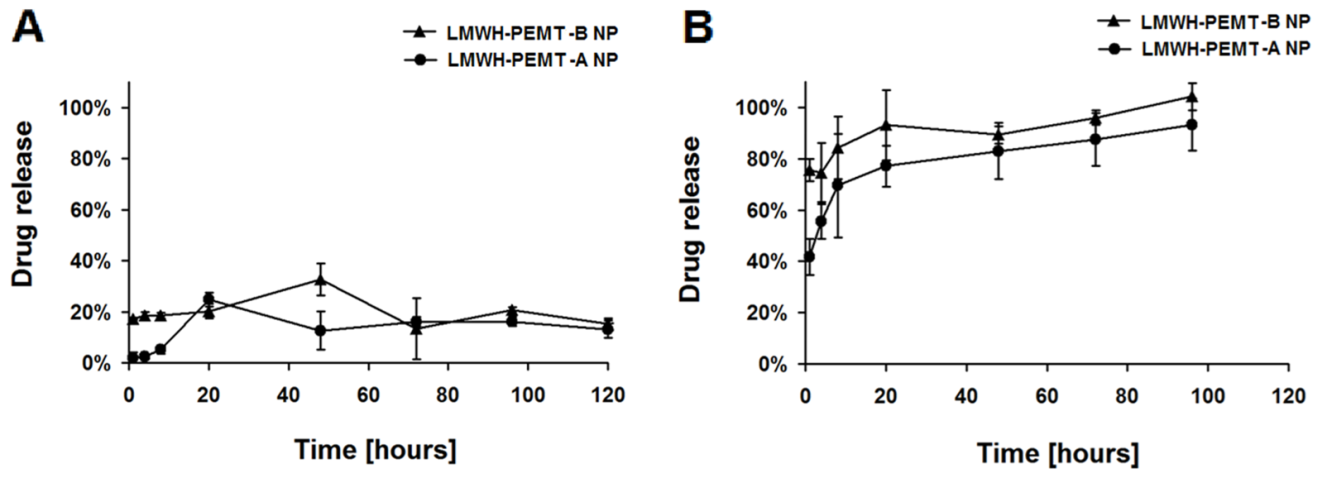
**

**Figure 3:** Cumulative LMWH release from cationic NP at +37^o^ C in buffered medium PH 7.4 (**A**), or in buffered medium with mucin 2.5% (**B**). (n=3, data are shown as mean ± S.D).

**
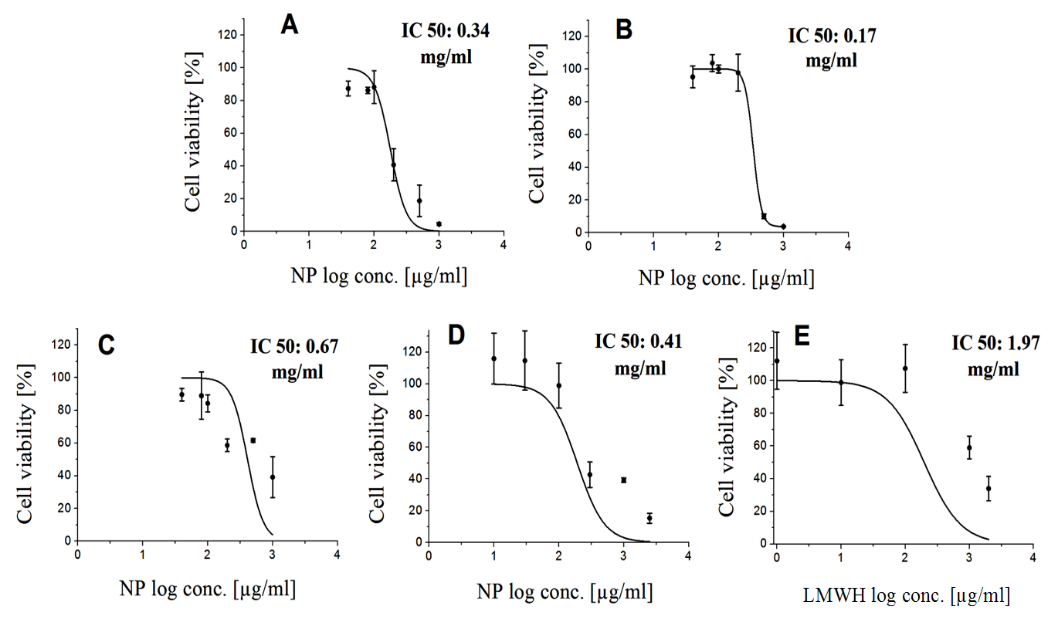
**

**Figure 4:** Macrophages viability (IC50) after 8 hours incubation with the drug and NP formulations. **A:** PEMT-A NP, **B:** PEMT-B NP, **C:** LMWH-PEMT-A NP, **D:** LMWH-PEMT-B NP and **E:** LMWH. n= 3, data represent mean SD.

**
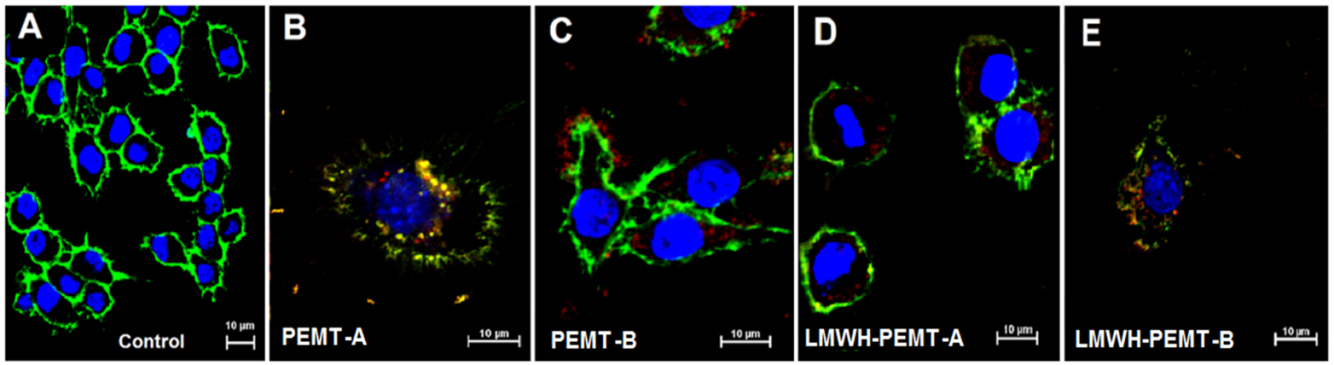
**

**Figure 5:** CLSM images of macrophages after 2 hours incubation with DiI-labelled NP. Cell membrane was stained green using wheat germ agglutinin-FITC, and the nucleus was stained blue with DAPI.


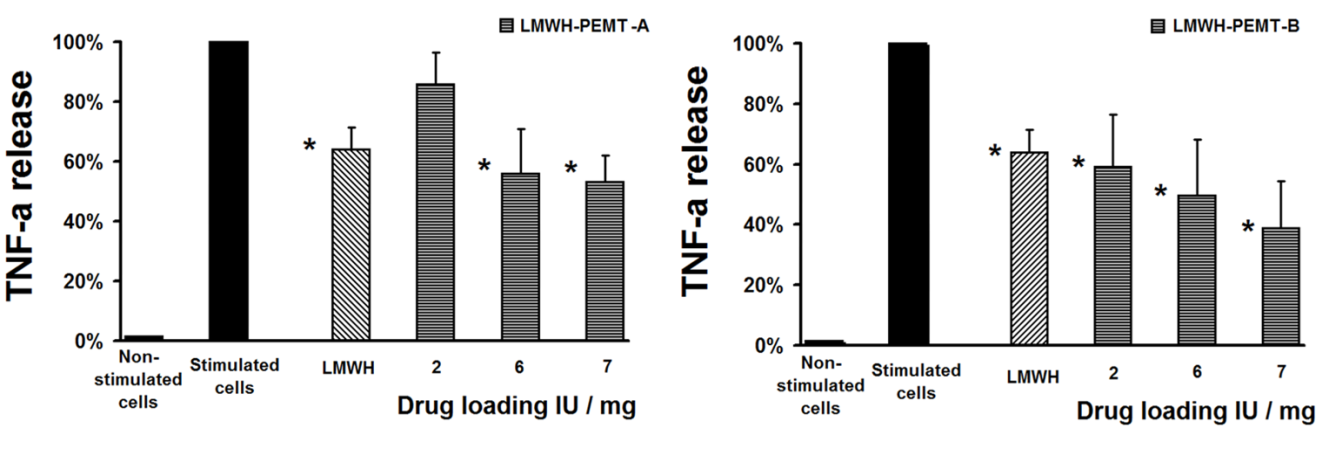


**Figure 6:** TNF-α release from LPS stimulated macrophages after 8 hours of incubation with LMWH-NP. NP were associated with increased amounts of LMWH and applied at concentration (100 µg/ml). LMWH was used at a concentration of 1 IU/ml. Results are expressed as [%] to stimulated cells without treatment. (n= 3, data represent mean SD). * = P<0.05 compared to stimulated cells.


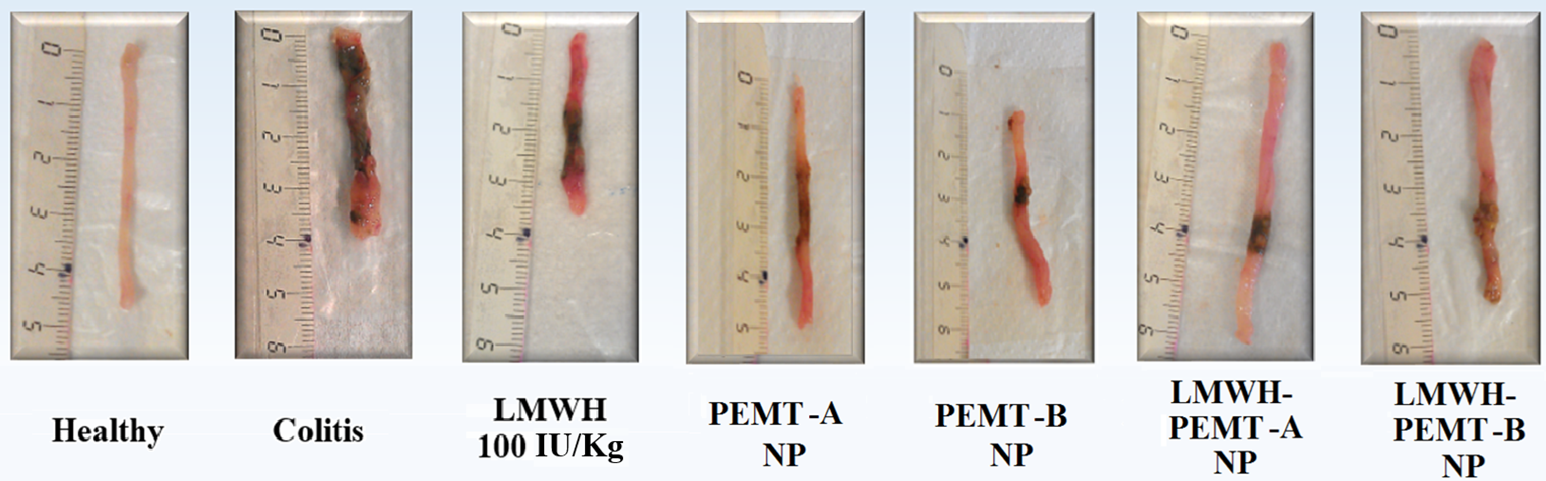


**Figure 7:** Colon tissues of mice treated with LMWH and LMWH-NP formulations. The dose of both the drug and drug loaded NP was equal to 100 IU /Kg. Abnormal tissue architecture and necrosis is shown in all treated groups in comparison to healthy group.


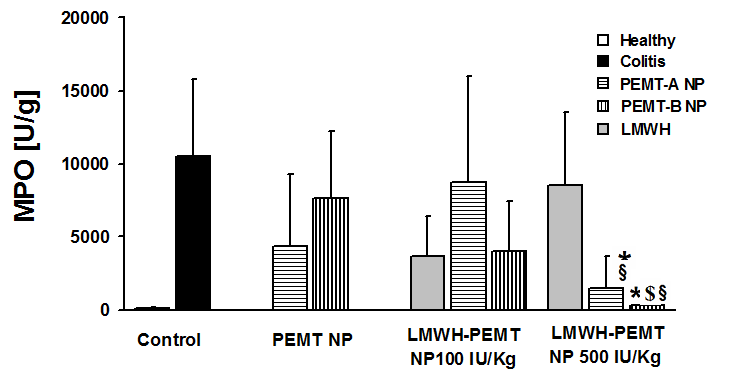


**Figure 8:** MPO activity in the colon tissue of the colitis model mice after treatment with NP formulations. Data are shown as mean ± S.D (n=5). Kruskal-Wallis ANOVA followed by tukey test were used for statistical analysis; *, $, § = P<0.05 compared with each of colitis control mice, blank NP and LMWH groups respectively. Only LMWH-NP (500 IU/Kg) were able to achieve an efficient reduction of MPO activity.
